# The Impacts of Different Types of Radiation on the CRT and PDL1 Expression in Tumor Cells Under Normoxia and Hypoxia

**DOI:** 10.3389/fonc.2020.01610

**Published:** 2020-08-19

**Authors:** Yangle Huang, Qingting Huang, Jingfang Zhao, Yuanli Dong, Lijia Zhang, Xumeng Fang, Pian Sun, Lin Kong, Jiade Jay Lu

**Affiliations:** ^1^Department of Radiation Oncology, Shanghai Proton and Heavy Ion Center, Fudan University Cancer Hospital, Shanghai, China; ^2^Shanghai Engineering Research Center of Proton and Heavy Ion Radiation Therapy, Shanghai, China; ^3^Department of Medical Physics, Shanghai Proton and Heavy Ion Center, Fudan University Cancer Hospital, Shanghai, China

**Keywords:** proton radiation, carbon-ion radiation, normoxia, hypoxia, calreticulin, PDL1

## Abstract

**Introduction:**

Hypoxia is a hallmark of cancer that may contribute to an immunosuppressive microenvironment and promote radioresistance. High linear energy transfer (LET) radiation is considered to be able to overcome the negative effects of hypoxia. However, the anti-tumorigenic effects induced by low or high LET radiation have not been fully elucidated. This study aimed to compare the effects of different types of radiation on the immune response, particularly the impact on calreticulin (CRT), and programmed cell death ligand 1 (PDL1) expression.

**Methods:**

Four human tumor cell lines were investigated in this study. Cells in normoxic and hypoxic groups were irradiated with 4Gy (physical dose) photon, proton, and carbon-ion radiation, respectively. The expression of CRT and PDL1 was detected 48 h after irradiation, and the median fluorescence intensities (MFIs) were compared by flow cytometry. Meanwhile, the radiosensitivity of tumor cells in each group was also compared by colony formation assays and flow cytometry.

**Results:**

All types of radiation could significantly inhibit the colony formation of tumor cells under normoxia. However, the efficacy of photon and proton radiation was impaired under hypoxia. Carbon-ion radiation could still inhibit colony formation. The percentage of viable cells after irradiation was higher under hypoxia compared with those under normoxia. The CRT expression under normoxia was significantly increased after radiation. Carbon-ion radiation enhanced CRT expression compared to photon and proton radiation. Conversely, under hypoxia, the CRT expression level was significantly upregulated at baseline (0Gy). Radiation could not increase the expression further. PDL1 expression was also significantly increased by radiation under normoxia in all cell lines except the Ln18 cell line. Carbon-ion radiation induced the most significant increase. Under hypoxia, the PDL1 expression level was also upregulated at baseline and radiation could not increase expression further.

**Conclusion:**

Tumor cells were resistant to photon and proton but sensitive to carbon-ion radiation under hypoxia. Carbon-ion radiation could induce the highest CRT and PDL1 expression under normoxia. However, under hypoxia, radiation could not further enhance the high baseline expression of CRT and PDL1.

## Introduction

Hypoxia is one of the hallmarks of malignant tumors (1). Tumors under hypoxia are more aggressive than those under normoxia, which is characterized by a higher rate of metastasis and increased resistance to chemo- and radiotherapy (2, 3). Thus, hypoxia is considered an unfavorable prognostic factor for various malignant tumors, especially inoperable head and neck cancers (4). Though many hypoxia-targeting strategies have been investigated in clinical research, they have been ineffective (5). Additionally, hypoxia can contribute to the immune escape of tumor cells via the upregulation of programmed cell death ligand 1 (PDL1) in a hypoxia-inducible factor-1α (HIF-1α)-dependent manner (6). Thus, the anti-tumor effects exerted by the immune system following radiation would be reduced in an immunosuppressive hypoxic environment.

It is widely acknowledged that the cytotoxic effects of radiation are predominantly due to the damage of DNA in cells. DNA damage can be caused by both the direct and indirect effects of radiation. For low linear energy transfer (LET) radiation, like photon, DNA is damaged indirectly via free radicals, while for high LET radiation, like carbon-ion, DNA is ionized, and damaged directly (7). Free radicals react with DNA to form superoxide in the presence of molecular oxygen, which results in DNA damage. The absence of oxygen would therefore decrease DNA damage mediated by radicals (8). However, the direct effect of radiation is independent of oxygen. Thus, the contribution of oxygen is likely different between the low and high LET radiation. Previous studies have shown that the oxygen enhancement ratio (OER) for photon radiation is 2.5–3.5, while for carbon-ion radiation, the OER is closer to 1–1.5 (9). Therefore, carbon-ion radiation is considered able to overcome the unfavorable effect of hypoxia on radiotherapy, at least to some extent.

Carbon-ion and proton radiation are the most advanced techniques used in clinical practice. They have radio-biological and radio-physical advantages over conventional photon radiation. However, the anti-tumor effects induced by proton and carbon-ion radiation have not been fully elucidated. Increased translocation of calreticulin (CRT) to the surface of cell membrane occurs when cells undergo immunogenic cell death, and ecto-CRT has been shown to play an important role in adaptive immune response (10). We previously compared the impact of photon, proton, and carbon-ion radiation on CRT expression in normoxic conditions (11). The impacts of different types of radiation on CRT and PDL1 expression under hypoxia are still poorly understood. Thus, our aim was to compare the effects of photon, proton, and carbon-ion radiation on the expression of CRT and PDL1 under normoxia and hypoxia. This study provided important information and improved our understanding of the anti-tumorigenic responses induced by radiotherapy, especially proton and carbon-ion radiation.

## Materials and Methods

### Cell Lines and Culture Conditions

Four human tumor cell lines were investigated in this study. These included tongue squamous carcinoma cell lines Tca8113 and Cal27, and the glioma cell lines Ln229 and Ln18. All cells were cultured in DMEM medium containing 10% fetal bovine serum (FBS) supplemented with 1% streptomycin and penicillin. Tumor cells in the normoxic group were cultured in an incubator at 37°C containing 5% CO_2_ and 21% O_2_, while cells in the hypoxic group were cultured in a hypoxic chamber at 37°C containing 5% CO_2_ and 0.5% O_2_.

### Irradiation

Exponentially growing tumor cells were irradiated with photon, proton, or carbon-ion radiation as previously described (11). The LET value of photon, proton, and carbon-ion radiation was 2.00, 1.98, and 29.14 keV/μm, respectively. The irradiation doses mentioned are physical doses. Cells in the normoxic group were exposed to radiation directly. While cells in the hypoxic group were placed in a hypoxic culture bag (AnaeroPack, Mitsubishi Gas Chemical Company) in advance, to ensure that tumor cells were in hypoxic condition during the radiation. After irradiation, cells from all the groups, including the mock-irradiated control group (0Gy), were washed twice with phosphate buffer saline (PBS), and the culture medium was replaced. Cells were then immediately cultured in normoxic or hypoxic condition as mentioned above.

### Colony Formation Assay

After irradiation with 4Gy of photon, proton, or carbon-ion radiation, the tumor cells in both the normoxic and hypoxic groups were immediately trypsinized and evenly seeded (5000 cells per well) in six-well plates. Three independent experiments were performed for each group. Cells were then cultured in normoxic or hypoxic conditions to form colonies. Colonies were fixed with methanol and stained with crystal violet after 7 days. Images of each group were captured by a colony counting machine (GelCount, Oxford Optronix Ltd.). Only those containing more than 50 cell colonies were counted. The survival fraction (SF) of tumor cells was calculated as follows: colony formation rate in the irradiating group/colony formation rate in the control group.

### Flow Cytometry Analysis of Live and Dead Cells

Tumor cells were cultured for 48 h following irradiation with different types of radiation under normoxia or hypoxia. Tumor cells in each group, including the control group (0Gy), were washed with PBS and harvested using trypsin solution without EDTA. Cells were double stained with PE/Annexin V and 7-Amino-Actinomycin (7AAD; Apoptosis detection kit, BD Pharmingen, 559763) according to the manufacturer’s instructions. Samples of each group were examined by flow cytometry (CytoFLEX S, Beckman Coulter), and the results were analyzed by CytoExpert software (version 2.3, Beckman Coulter).

### Flow Cytometry Analysis of CRT and PDL1 Expression

Tumor cells were harvested 48 h after irradiation. Each sample was incubated in blocking buffer (PBS containing 5% FBS) for 15 min, followed by washing with cold PBS. Tumor cells were then incubated with P-phycoerythrin (PE) conjugated anti-CRT (PE-CRT, Abcam, and ab83220) or anti-PDL1 (PE-PDL1, CST, and 71391) monoclonal antibodies, respectively. The fluorescence intensity of CRT and PDL1 in each group was detected on a flow cytometer (CytoFLEX S, Beckman Coulter). Flow cytometry results were analyzed by FlowJo (version 10.0.7, Three Star, Inc). The median fluorescence intensity (MFI) was compared between irradiated groups and the non-irradiated group (control group). The fold change of MFI was used to compare the expression of CRT and PDL1 among different groups.

### Statistical Analysis

Statistical analysis was conducted by GraphPad Prism (version 7.0, GraphPad Software). Unpaired Student’s *t* test was used to test the significant difference between two independent samples. Two-way ANOVA was used to test the significant difference between two independent groups. *P* value < 0.05 was considered statistically significant.

## Results

### Comparison of the Inhibitory Effects on Colony Formation by Photon, Proton, and Carbon-Ion Radiation Under Normoxia and Hypoxia

**FIGURE 1:**
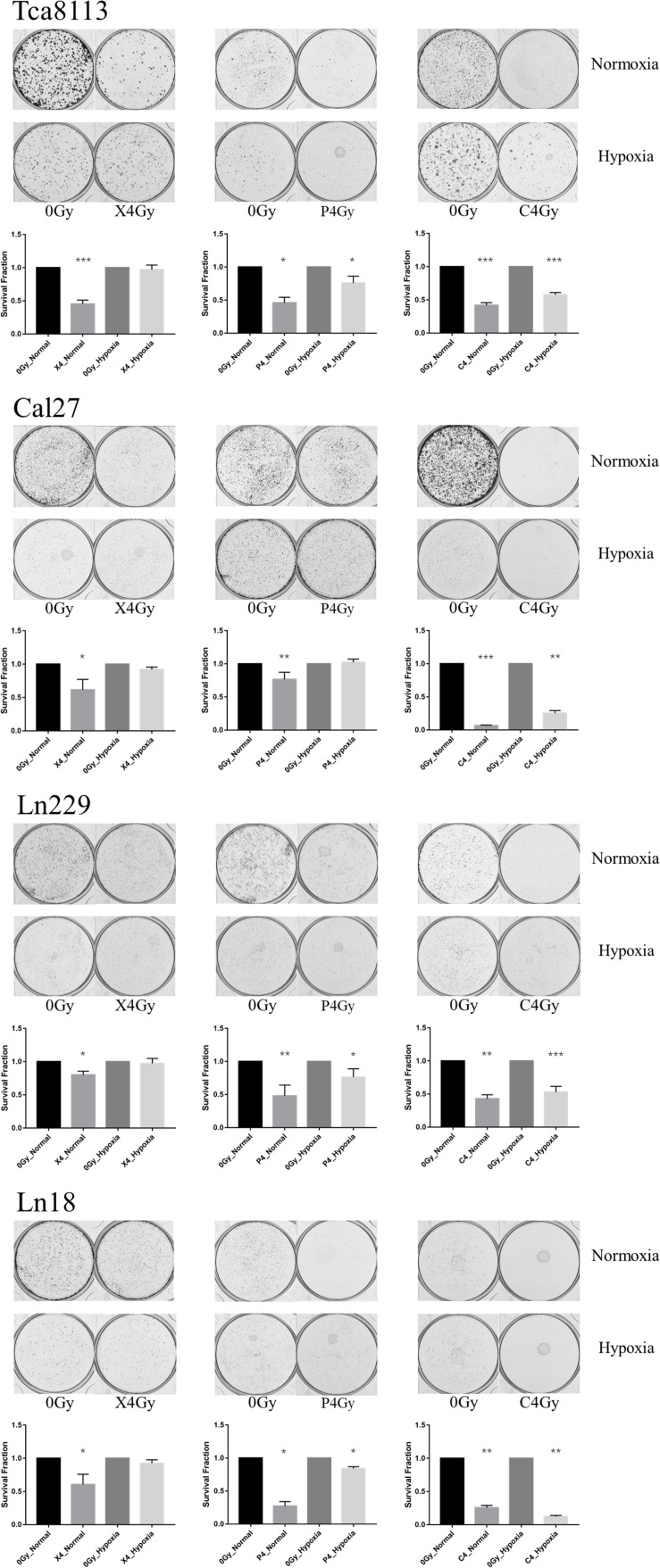
Comparison of the inhibitory effects on colony formation by the three types of radiation under normoxia and hypoxia. Tumor cells were irradiated with 4Gy (physical dose) photon, proton, or carbon-ion radiation under normoxia or hypoxia conditions. Survival fraction (SF) was calculated as: colony formation rate in the irradiated group/colony formation rate in the control group. The representative images of colony formation for each tumor cell group are shown in the upper panels. The histograms indicate the SF of tumor cells exposed to different types of radiation under normoxic and hypoxic conditions. Results are presented as mean ± s.d. Statistical significance of each irradiated group relative to the corresponding control group (0Gy) was indicated by asterisks. *p < 0.05, **p < 0.01, and ***p < 0.001.

Four tumor cell lines were irradiated with 4Gy (physical dose) photon, proton, or carbon-ion radiation under normoxic or hypoxic conditions. Cells were trypsinized immediately after irradiation and cultured in six-well plates (5000 cells/well). Mock-irradiated groups (0Gy) under normoxia and hypoxia were cultured like controls. After culture for 7–11 days, cells were fixed and stained with crystal violet. The SF and the representative images of colony formation for each group are shown in Figure 1. The SF of each cell line under normoxia or hypoxia following different types of radiation is shown in Table 1.

**TABLE 1:** The survival fraction of tumor cells following irradiation under normoxia and hypoxia.

**Tumor cell**	**Normoxia group**	**SF**	**95% CI**	**Hypoxia group**	**SF**	**95% CI**
Tca8113	X4	0.45	0.30–0.60	X4	0.97	0.80–1.14
Tca8113	P4	0.46	0.25–0.67	P4	0.76	0.50–1.02
Tca8113	C4	0.42	0.32–0.52	C4	0.58	0.49–0.66
Cal27	X4	0.62	0.23–1.00	X4	0.91	0.83–1.01
Cal27	P4	0.76	0.50–1.04	P4	1.02	0.90–1.15
Cal27	C4	0.06	0.04–0.09	C4	0.25	0.16–0.35
Ln229	X4	0.80	0.66–0.90	X4	0.97	0.79–1.16
Ln229	P4	0.48	0.07–0.89	P4	0.76	0.45–1.08
Ln229	C4	0.43	0.28–0.58	C4	0.53	0.31–0.75
Ln18	X4	0.60	0.22–0.99	X4	0.93	0.80–1.05
Ln18	P4	0.27	0.10–0.44	P4	0.84	0.77–0.91
Ln18	C4	0.26	0.17–0.34	C4	0.12	0.08–0.16

*SF, survival fraction.*

According to the results above, photon, proton, and carbon-ion radiation could all inhibit colony formation of tumor cells under normoxia. However, the SF of the photon and proton radiation groups under hypoxia was not significantly reduced. Conversely, carbon-ion radiation significantly reduced the SF in hypoxic conditions. These results suggested that the ability of photon and proton radiation to inhibit tumor cell colony formation was weakened under hypoxia, while carbon-ion radiation still possessed solid inhibitory effects under hypoxia. Therefore, carbon-ion radiation was less affected by hypoxia when compared to photon and proton irradiation.

### Comparison Between the Percentage of Viable and Dead Tumor Cells After Photon, Proton, and Carbon-Ion Radiation Under Normoxia and Hypoxia

**FIGURE 2:**
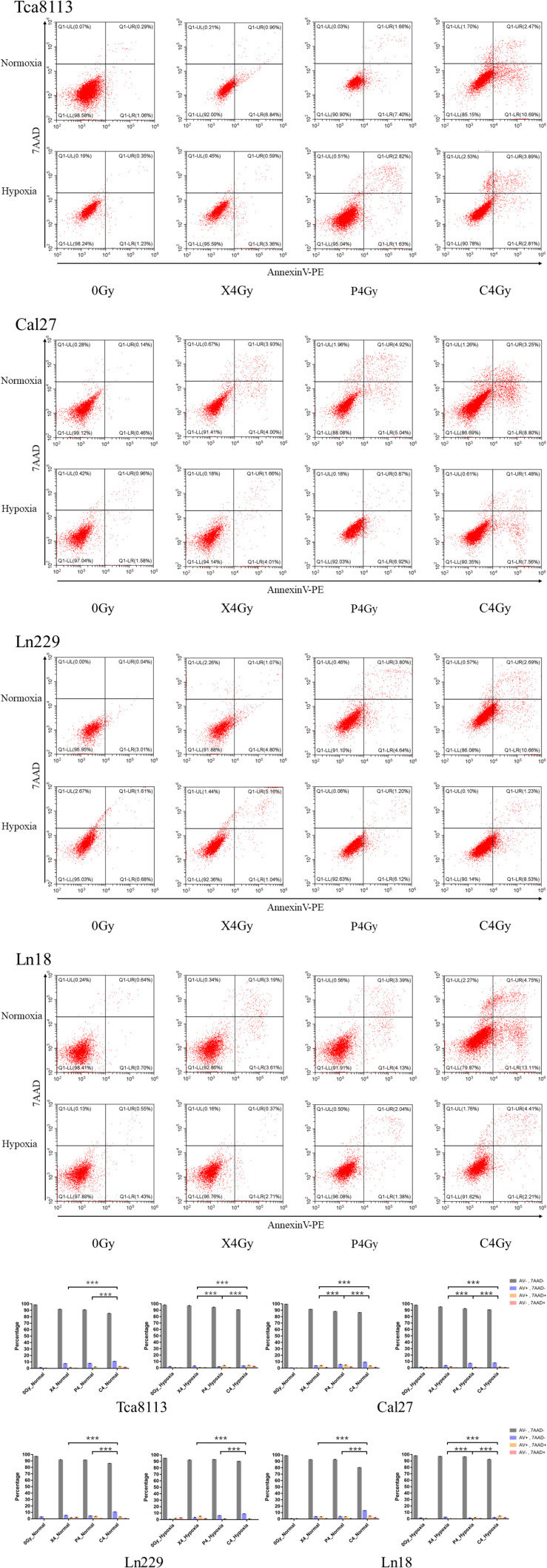
Comparison between the percentage of viable and dead cells under normoxia and hypoxia. Tumor cells in normoxic and hypoxic conditions were exposed to 4Gy physical dose photon, proton, or carbon-ion radiation. Cell survival was detected 48 h after irradiation using the Annexin V/7-AAD double staining kit. Representative flow cytometry images for each group are shown in the scatter plots. Statistical analysis of the cell survival and death percentages for each group are shown in the histograms. Each experiment was repeated at least three times. ***p < 0.001.

In order to compare the percentage of viable and dead tumor cells 48 h after exposure to different types of radiation under normoxia and hypoxia, we treated tumor cells with 4Gy physical dose photon, proton, and carbon-ion radiation under normoxic and hypoxic conditions. Irradiated cell groups, in addition to mock-irradiated control groups (0Gy), were cultured under the same oxygen conditions for 48 h. Next, we used Annexin V/7-AAD to detect viable and dead cells by flow cytometry. Cells that were Annexin V-negative and 7-AAD-negative (AV-/7AAD-) were considered viable. The percent viability of tumor cells in each group are shown in Table 2. Annexin V-positive and 7-AAD-negative (AV+/7AAD-) cells were considered to be in early apoptosis, while Annexin V and 7-AAD positivity (AV+/7AAD+) suggested that cells were in late apoptosis or dead. Representative flow cytometry images for each group and the percentages of viable and dead cells are shown in Figure 2.

**TABLE 2:** The percentage of viable tumor cells in each group 48 h after irradiation under normoxia and hypoxia.

**Tumor cell**	**Normoxia group**	**Survival (%)**	**95% CI**	**Hypoxia group**	**Survival (%)**	**95% CI**
Tca8113	0Gy	98.39	97.96–98.81	0Gy	97.61	95.43–99.79
Tca8113	X4	91.34	89.91–92.77	X4	96.57	94.34–98.80
Tca8113	P4	90.38	88.75–92.01	P4	94.22	92.34–96.10
Tca8113	C4	84.81	83.19–86.44	C4	90.63	90.22–91.05
Cal27	0Gy	99.29	98.86–99.71	0Gy	97.64	96.35–98.93
Cal27	X4	91.37	91.06–91.68	X4	94.82	93.33–96.32
Cal27	P4	87.78	86.38–89.17	P4	92.06	90.38–93.74
Cal27	C4	86.31	85.49–87.13	C4	90.52	90.03–91.00
Ln229	0Gy	97.04	96.53–97.54	0Gy	94.80	94.17–95.43
Ln229	X4	91.37	89.66–93.08	X4	91.84	90.05–93.63
Ln229	P4	91.34	90.82–91.85	P4	92.78	92.39–93.17
Ln229	C4	85.99	84.95–87.02	C4	90.02	89.59–90.45
Ln18	0Gy	98.7	98.08–99.31	0Gy	98.06	97.69–98.44
Ln18	X4	92.33	91.05–93.61	X4	97.00	96.43–97.58
Ln18	P4	92.39	90.38–94.41	P4	96.29	95.53–97.05
Ln18	C4	80.17	79.51–80.83	C4	92.05	90.45–93.65

The percentage of viable tumor cells was all increased under hypoxia in comparison to normoxia following irradiation with photon, proton, or carbon-ion radiation, which suggests that tumor cells were more resistant to radiation in hypoxic conditions. Carbon-ion radiation was capable of inducing more cell death compared with photon or proton radiation at the same physical dose while cells were hypoxic.

### Comparison of Tumor Cell CRT Expression Under Normoxia and Hypoxia in Each Group

**FIGURE 3:**
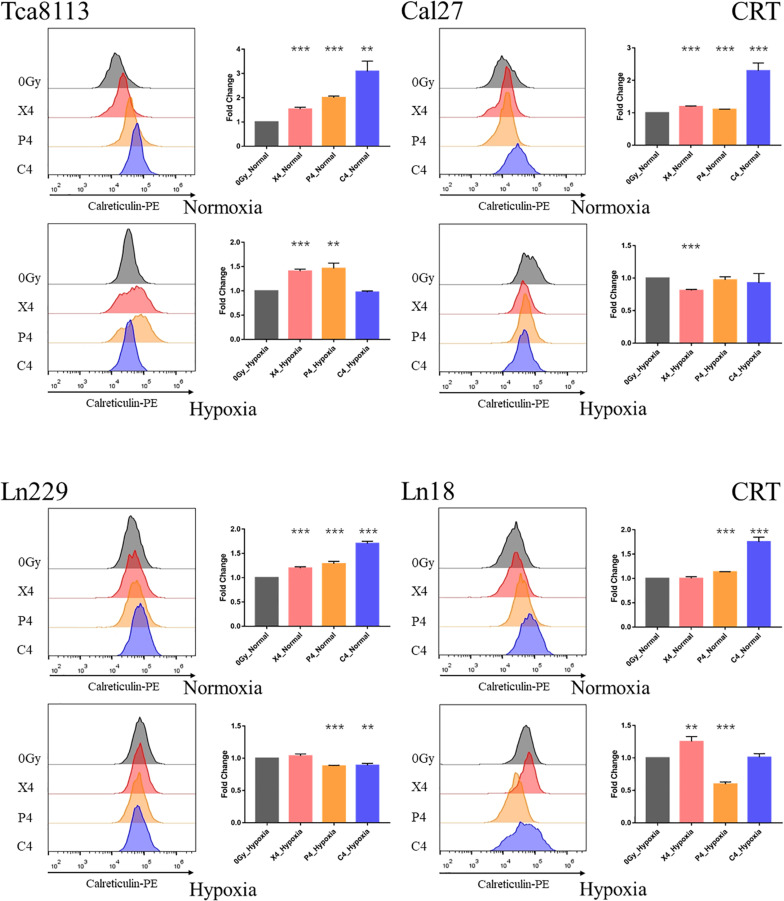
Comparison of CRT expression under normoxia and hypoxia. Tumor cells were exposed to 4Gy physical dose photon, proton, or carbon-ion radiation. The expression level of CRT on the tumor cell surface was detected by flow cytometry 48 h after irradiation. Representative flow cytometry images for each group are shown in the half-offset histograms. The horizontal axis represents the fluorescence intensity of CRT-PE, and the vertical axis represents the number of cells. The fold change of the median fluorescence intensity (MFI) for each group relative to the control group (0Gy) is shown in the bar charts. Results are presented as mean ± s.d. Each experiment was repeated at least three times. Statistical significance of each irradiated group relative to the control group (0Gy) was indicated by asterisks. **p < 0.01, and ***p < 0.001.

Next, we compared the changes in expression of CRT on the tumor cell membrane 48 h after irradiation with 4Gy physical dose photon (X4), proton (P4), or carbon-ion (C4) radiation compared to the control group (0Gy) under normoxia and hypoxia. The MFI of CRT staining was detected by flow cytometry for each group. Representative flow cytometry images and statistical significance are demonstrated in Figure 3.

The fold change of CRT expression in each irradiation group compared to the control group under normoxia and hypoxia are listed in Table 3.

**TABLE 3:** The changes in CRT expression for each irradiation group under normoxia and hypoxia.

**Irradiating group (normoxia)**	**Fold change**	**95% CI**	**Irradiating group (hypoxia)**	**Fold change**	**95% CI**
Tca8113_X4	1.53	1.35–1.71	Tca8113_X4	1.41	1.30–1.51
Tca8113_P4	2.00	1.84–2.17	Tca8113_P4	1.46	1.19–1.74
Tca8113_C4	3.09	2.02–4.15	Tca8113_C4	0.97	0.91–1.04
Cal27_X4	1.19	1.13–1.25	Cal27_X4	0.81	0.76–0.85
Cal27_P4	1.11	1.08–1.13	Cal27_P4	0.97	0.86–1.09
Cal27_C4	2.30	1.70–2.90	Cal27_C4	0.93	0.56–1.29
Ln229_X4	1.20	1.12–1.27	Ln229_X4	1.03	0.95–1.12
Ln229_P4	1.29	1.16–1.41	Ln229_P4	0.88	0.86–0.90
Ln229_C4	1.70	1.58–1.82	Ln229_C4	0.89	0.81–0.97
Ln18_X4	1.00	0.92–1.08	Ln18_X4	1.26	1.08–1.15
Ln18_P4	1.13	1.12–1.15	Ln18_P4	0.60	0.52–0.67
Ln18_C4	1.75	0.90–1.86	Ln18_C4	1.01	0.88–1.14

**FIGURE 4:**
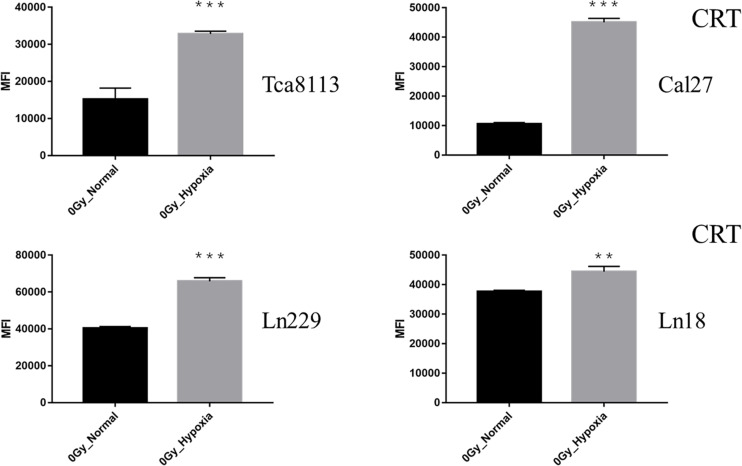
Comparison of baseline CRT expression under normoxia and hypoxia. The expression of CRT on the tumor cell surface was detected by flow cytometry. The MFI of each tumor cell line under normoxia and hypoxia is shown in the histogram. Results are presented as mean ± s.d. Each experiment was repeated at least three times. Statistical significances of the difference between cells under normoxia and hypoxia are indicated by asterisks. **p < 0.01, ***p < 0.001.

When comparing the CRT expression between normoxic and hypoxic cells at baseline (0Gy, control groups), all the tumor cells in the hypoxic groups expressed more CRT than the normoxic groups. As demonstrated in Figure 4, the CRT expression under hypoxia increased by 2.21-fold (95% CI: 1.33–3.09), 4.27-fold (95% CI: 3.90–4.63), 1.63-fold (95% CI: 1.58–1.68), and 1.18-fold (95% CI: 1.10–1.26) for Tca8113, Cal27, Ln229, and Ln18 cell lines, respectively.

These results indicated that photon, proton, and carbon-ion radiation could all significantly increase the expression of CRT on tumor cells in normoxic conditions. Carbon-ion radiation could induce more CRT expression compared to photon and proton radiation at the same physical dose. Alternatively, the CRT expression on tumor cells was upregulated at baseline (0Gy) in hypoxic conditions. In these hypoxic conditions, photon, proton, or carbon-ion radiation could not further increase CRT expression. In some radiation groups, CRT expression was decreased after radiation.

### Comparison of PDL1 Expression in Tumor Cells Under Normoxia and Hypoxia Following Irradiation

**FIGURE 5:**
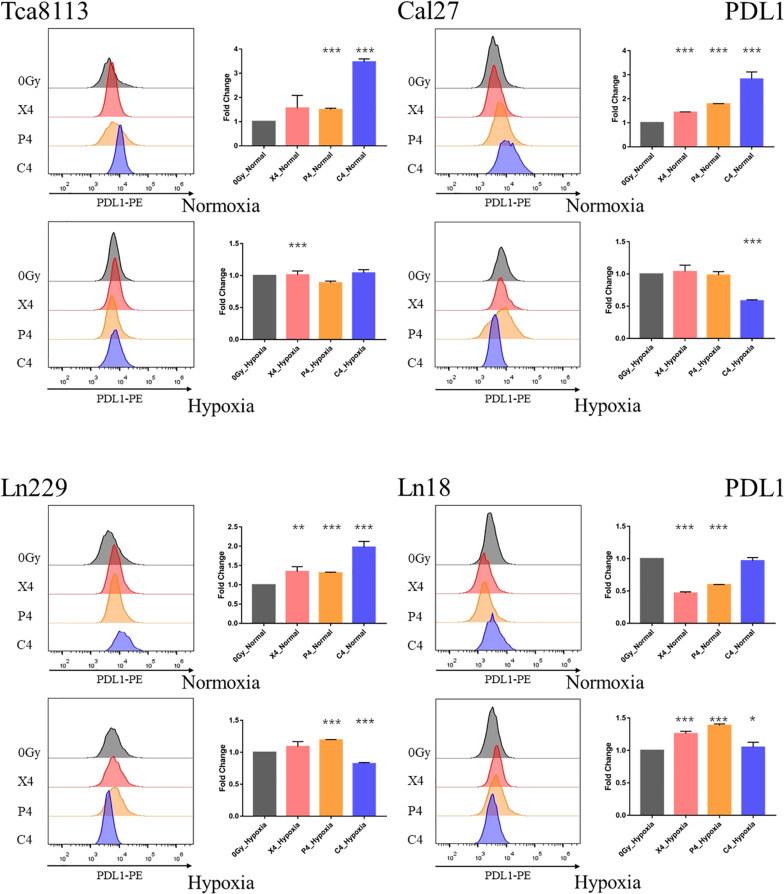
Comparison of PDL1 expression under normoxia and hypoxia. Tumor cells were exposed to 4Gy physical dose photon, proton, or carbon-ion radiation. The expression level of PDL1 on the tumor cell surface was detected by flow cytometry 48 h after irradiation. Representative flow cytometry images of each group are shown in the half-offset histograms. The horizontal axis represents the fluorescence intensity of PDL1-PE, and the vertical axis represents the number of cells. The fold change of the MFI for each group relative to the control group (0Gy) is shown in the bar charts. Results are presented as mean ± s.d. Each experiment was repeated at least three times. Statistical significance of each irradiated group relative to the control group (0Gy) was indicated by asterisks. *p < 0.05, **p < 0.01, and ***p < 0.001.

We compared the changes in PDL1 expression on tumor cell membranes 48 h after exposure to 4Gy physical dose photon (X4), proton (P4), or carbon-ion (C4) radiation under normoxia or hypoxia. The MFI of PDL1 was also detected by flow cytometry in each group. Representative flow cytometry images and statistical significance are demonstrated in Figure 5.

The fold change of PDL1 expression in each irradiation group compared to the control group under normoxia and hypoxia are listed in Table 4.

**TABLE 4:** The changes in PDL1 expression for each irradiation group under normoxia and hypoxia.

**Irradiating group (normoxia)**	**Fold change**	**95% CI**	**Irradiating group (hypoxia)**	**Fold change**	**95% CI**
Tca8113_X4	1.57	0.74–2.40	Tca8113_X4	1.01	0.95–1.08
Tca8113_P4	1.50	1.34–1.65	Tca8113_P4	0.89	0.84–0.93
Tca8113_C4	3.47	3.16–3.78	Tca8113_C4	1.04	0.98–1.11
Cal27_X4	1.44	1.41–1.48	Cal27_X4	1.04	0.93–1.14
Cal27_P4	1.79	1.77–1.80	Cal27_P4	0.98	0.85–1.12
Cal27_C4	2.82	2.45–3.19	Cal27_C4	0.58	0.55–0.61
Ln229_X4	1.35	1.22–1.48	Ln229_X4	1.09	1.01–1.17
Ln229_P4	1.31	1.26–1.36	Ln229_P4	1.19	1.18–1.21
Ln229_C4	1.97	1.60–2.38	Ln229_C4	0.82	0.78–0.87
Ln18_X4	0.47	0.42–0.52	Ln18_X4	1.26	1.17–1.35
Ln18_P4	0.60	0.59–0.61	Ln18_P4	1.39	1.36–1.41
Ln18_C4	0.97	0.84–1.09	Ln18_C4	1.05	0.95–1.15

**FIGURE 6:**
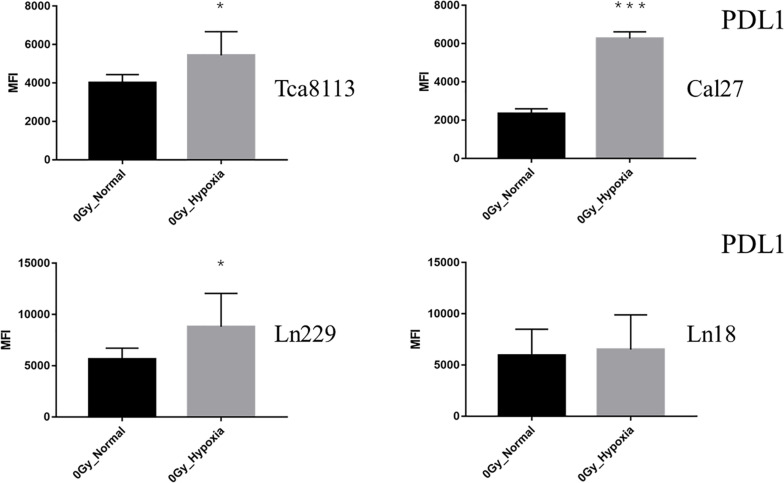
Comparison of baseline PDL1 expression under normoxia and hypoxia. The expression of PDL1 on the tumor cell surface was detected by flow cytometry. The MFI of each tumor cell line under normoxia and hypoxia is shown in the histogram. Results are presented as mean ± s.d. Each experiment was repeated at least three times. Statistical significance of the difference between cells under normoxia and hypoxia are indicated by asterisks. *p < 0.05, ***p < 0.001.

The baseline PDL1 expression (0Gy, control group) of all tumor cell lines in hypoxic conditions was upregulated in comparison to those in the normoxic group. As shown in Figure 6, the PDL1 expression under hypoxia increased by 2.64-fold (95% CI: 2.04–3.25), 1.36-fold (95% CI: 0.84–1.89), 1.50-fold (95% CI: 1.03–1.98), and 1.28-fold (95% CI: 0.53–2.04) for Tca8113, Cal27, Ln229, and Ln18 cell lines, respectively.

These results indicated that all types of radiation could increase the expression of PDL1 in all tumor cell lines except the Ln18 cell line under normoxic conditions, while under hypoxic conditions, the expression of PDL1 on tumor cells was upregulated at baseline (0Gy). Under these conditions, photon, proton, or carbon-ion radiation could not further increase PDL1 expression. Carbon-ion radiation could increase PDL1 expression more effectively than photon or proton radiation at the same physical dose under normoxia, but not under hypoxia. In some tumor cell lines, like Cal27 and Ln229, PDL1 expression may even be downregulated after exposure to carbon-ion radiation.

## Discussion

Oxygen plays an important role in the tumor response to radiotherapy, and the oxygenation profile of tumors tends to be very heterogeneous. Some tumors are well oxygenated while others are hypoxic (2). Even in different regions of a tumor, the oxygen concentration can be quite different (12). Thus, there can be normoxic and hypoxic regions within the tumor mass, and the extent of hypoxia varies. However, even a small amount of oxygen can be significant. When the oxygen concentration reaches 2%, the dose–response curve of the cell to radiation is no different from that observed in normoxic conditions. When the oxygen concentration is about 0.5%, the radiosensitivity of the cell is about half that in well-oxygenated conditions (7). Therefore, in this study, we used 0.5% oxygen concentrations to simulate the hypoxic environment in tumors. The normoxic group was exposed to around 21% oxygen. Here, we compared the expression of anti-tumor immunity-related molecules, CRT and PDL1, in response to different types of radiation in normoxic and hypoxic conditions.

The first section of this study compared the radioresistance of tumor cells in normoxic and hypoxic conditions to photon, proton, or carbon-ion radiation exposure. Colony formation assays and analysis of apoptosis indicated that tumor cells were significantly resistant to photon and proton radiation, although carbon-ion radiation still displayed effective cytotoxic effects toward tumor cells under hypoxia. In comparison to low LET radiation, like photon and proton, the cytotoxic effect of high LET radiation, like carbon ion, was less affected by hypoxic conditions. These results were consistent with previous studies concerning the OER of photon, proton, and carbon-ion radiation (9). As the OER value of carbon-ion radiation is lower than that of photon and proton radiation (1–1.5 vs. 2–3), the biological effects of carbon-ion radiation were not greatly affected by the oxygenation conditions. In addition to the difference in ionization effects (direct effect vs. indirect effect), different types of radiation can also produce different effects on the expression of radioresistance-related genes, like HIF1α. Worn et al. demonstrated that photon radiation could significantly upregulate HIF1α expression, while carbon-ion radiation did not induce increased HIF1α expression (13). HIF1α, as an important transcription factor, is involved in the expression of a series of downstream genes, such as vascular endothelial growth factor (VEGF). Thus, inhibiting HIF1α expression could significantly enhance the radiosensitivity of tumor cells (14, 15). In hypoxic conditions, there may be a synergistic effect between radiation and hypoxia on inducing HIF1α expression. Thus, the discrepant impacts on HIF1α upregulation by different types of radiation might partly affect the cytotoxic efficacy of radiotherapy, especially under hypoxia.

Photon, proton, and carbon-ion radiation could increase the expression of CRT in all four tumor cell lines in normoxic conditions. Consistent with our previous study, carbon-ion radiation could increase CRT expression compared with photon and proton radiation at the same physical dose (4Gy) (11). These results indicated that carbon-ion radiation might be able to enhance immunogenic cell death and enhance anti-tumorigenic responses compared with photon and proton radiation. When under hypoxic conditions, we found that the baseline (0Gy, group) CRT expression levels in the four tumor cell lines were significantly increased compared with those under normoxia. The expression level was upregulated by 2.21- to 4.27-fold for tongue squamous carcinoma cell lines, and by 1.18- to 1.63-fold for glioma cell lines (all *p* < 0.05). This upregulation of CRT expression might result from endoplasmic reticulum stress (ER stress) induced by hypoxia (16). When under the pressure of ER stress, large amounts of CRT (which is originally located in the endoplasmic reticulum cavity) would translocate to the surface of the cell membrane. Radiation can also induce ER stress mediated by reactive oxygen species (ROS) (17). Based on this study, CRT expression was not further increased by radiation compared with the control group in hypoxic conditions. Even carbon-ion radiation could not further increase the expression of CRT expression under hypoxic conditions. This phenomenon suggested that there might be an overlapping effect between CRT expression during hypoxia and radiation, which were both mediated by ER stress. The pressure of hypoxia induced abundant CRT translocation to the surface of the cell membrane and therefore radiation could not increase this further.

Previous studies have revealed that radiation could upregulate PDL1 expression (18, 19). The impact of radiation on PDL1 expression was thought to be related to DNA double-strand breaks (DSB) and the process of DNA damage repair (DDR) (20). Inhibiting key pathways within DDR, such as BRCA2 and Ku70/80, could result in a significant increase in PDL1 expression. We showed that all types of radiation could increase the expression of PDL1 under normoxia but carbon-ion radiation was the most effective. These results might be explained by the fact that carbon-ion radiation is capable of inducing more DSBs at the same physical dose (21, 22). However, for glioma cell line LN18, the expression level of PDL1 was downregulated to some extent, rather than upregulated after exposure to radiation. This suggested that PDL1 expression induced by irradiation may have cell specificity. Different tumors, even different subtypes, might have distinct PDL1 expression patterns in response to radiation, because of the discrepancy between radiosensitivity and DDR capacity. While under hypoxia, we observed that the baseline PDL1 expression was increased compared to groups under normoxia. Barsoum et al. reported that hypoxia could increase PDL1 expression in tumor cells through the HIF1α pathway, which resulted in the immune escape of tumors (6). Our current research showed that PDL1 expression was not further upregulated after exposure to radiation in hypoxic condition. In some irradiation groups, the expression levels of PDL1 were even downregulated compared with the control group. This could be because tumor cells exhibited radiation resistance under hypoxia. As such, the extent of DNA damage caused by radiation was reduced. As discussed previously, DDR was related to PDL1 expression, which may reflect the observed results. Additionally, the DDR process of tumor cells will also be altered under hypoxia (23–25). In some hypoxic tumor cells, the expression of homologous recombination repair (HRR) pathway-related genes, such as RAD51 and BCRA1, will be downregulated (26, 27), while the expression of non-homologous end-joining (NHEJ) pathway-related genes, such as ATM and DNA-PKcs, will be upregulated (28, 29). Regulation of DDR-related gene expression will also affect the radiosensitivity of tumor cells (30). Furthermore, for low and high LET radiation, the importance of distinct DDR pathways, like HRR and NHEJ, in response to DNA damage might be different. This might be another reason that the expression of PDL1 was different after exposure to photon, proton, or carbon-ion radiation under hypoxia.

In conclusion, this study compared the impacts of different types of radiation on CRT and PDL1 expression under normoxia and hypoxia. We found that carbon-ion radiation could increase CRT and PDL1 expression compared with photon and proton radiation in normoxic conditions. However, under hypoxia, the baseline expression levels of CRT and PDL1 were upregulated. Under these conditions, radiation could not further increase CRT and PDL1 expression. However, the underlying mechanisms regulating expression of these proteins have not been fully elucidated. In order to explore a therapeutic strategy that can overcome the immunosuppressive environment of hypoxia and enhance radiation-induced anti-tumorigenic responses, further studies are warranted, especially for the effective combination of immunotherapy and modern radiotherapy techniques, like proton and carbon-ion radiation.

## Data Availability Statement

The original contributions presented in the study are included in the article/Supplementary Material, further inquiries can be directed to the corresponding author/s.

## Author Contributions

JL and LK: conception and design, and administrative support. JZ, YH, and LZ: provision of study materials. YH, YD, QH, XF, and PS: collection and assembly of data. YH and YD: data analysis and interpretation. YH, LK, and JL: manuscript writing. All authors: final approval of manuscript.

## Conflict of Interest

The authors declare that the research was conducted in the absence of any commercial or financial relationships that could be construed as a potential conflict of interest.
