# Supplementary material for: The Impacts of Different Types of Radiation on the CRT and PDL1 Expression in Tumor Cells Under Normoxia and Hypoxia
Source: Front Oncol. 2020 Aug 19;10:1610. doi: 10.3389/fonc.2020.01610 (PMC7466457; doi:10.3389/fonc.2020.01610)
Supplement: Supplementary file 1 [file Data_Sheet_1.DOCX]

**
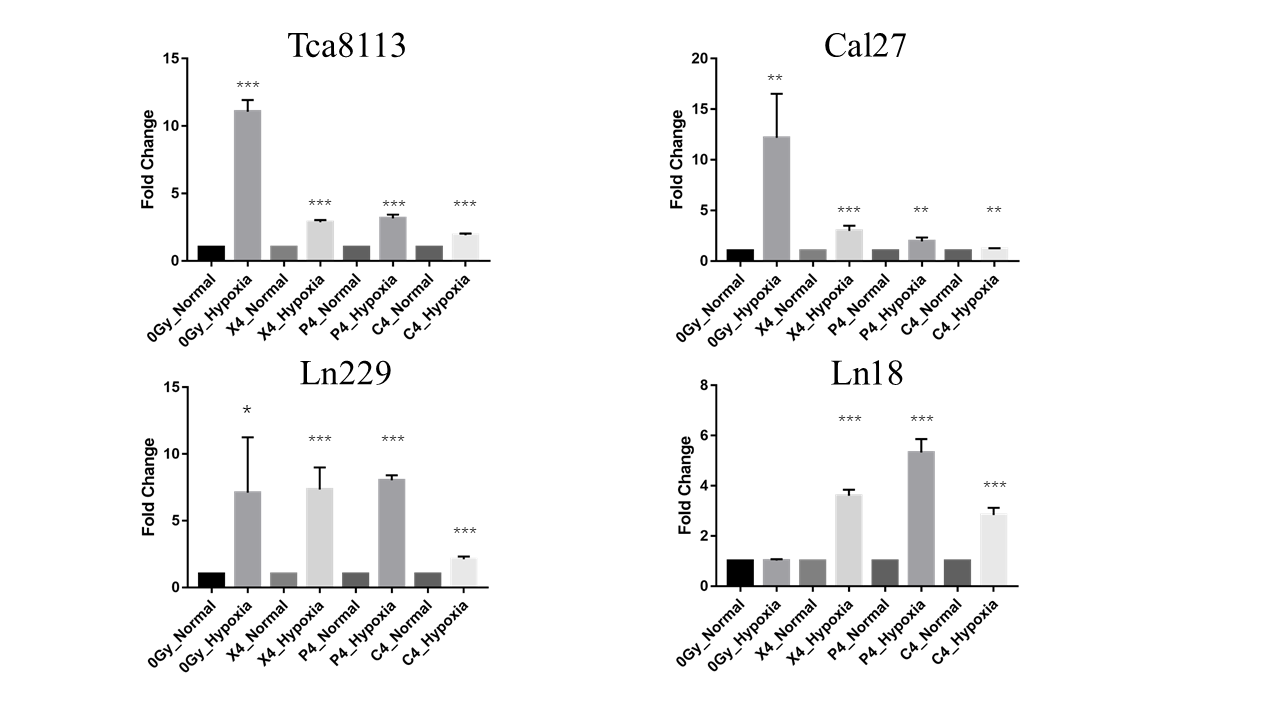
Supplementary Figure 1.** Comparison of the expression levels of VEGF between normoxia and hypoxia groups. Quantitative real-time PCR (qPCR) was used to compare the relative change of VEGF gene expression in each tumor cells between and normal and hypoxic groups. 0Gy represented the control group (mock-irradiated), X4 represented 4Gy photon (X-ray) radiation groups, P4 represented 4Gy proton radiation groups, and C4 represented 4Gy carbon-ion radiation groups. All the above-mentioned doses were physical dose. The vertical axis represented the fold change of VEGF expression in the hypoxia group relative to the corresponding normoxic group. Asterisks indicated the statistical significance of the hypoxic groups in relative to the normal groups. * indicated p<0.05, ** indicated p<0.01, *** indicated p<0.001, and none markers indicated p≥0.05.
